# The Effects of Alcohol

**Published:** 1905-05-13

**Authors:** 


The Hospital.

A Journal of

The Medical Sciences and Hospital Administration.

Vol. XXXVIII.—No. 972. SATURDAY, MAY 13, 1905.

### The Effects of Alcohol.

If we may assume that the human race would in many ways derive great benefit from the general ordering of life upon scientific principles, and that in this way health might be maintained in a degree hitherto almost unthought of, and disease almost abolished, we shall be entitled to scrutinise all the more closely the claims which are put forward in the name of science, and the character of the guidance which is offered to us on her behalf. When this guidance is incomplete or misleading, or when, as sometimes happens, it has no better foundation than an apparent claim to omniscience on the part of some distinguished person, it is only too often calculated to bring science into disrepute, rather than to produce a general willingness to be governed by her dictates. When Mr. Gladstone was at the height of his popularity it was the custom of his followers to hang reverently upon his lips for precepts concerning the domestic manufacture of jam; although people of calm judgment would have been more likely to rely for this purpose upon the teaching of an experienced housekeeper or stillroom maid. Somewhat in the same manner, a very distinguished and capable surgeon has been giving to the world his opinions concerning the use of alcohol as an article of diet, and, according to the reports of his discourse, has been classing it as a poison with arsenic, opium, and strychnia. He has brought about the very natural result that, when his views were quoted in the House of Commons as authoritative, the Lord Advocate declared them to be "too extreme for any reasonable man to accept." The mischief of such extreme pronouncements is that they are in excess of known and proven truth concerning the subjects to which they relate; and hence, like other forms of exaggeration, they are liable to defeat themselves. They even do more than this; they tend to bring real knowledge into disrepute. Nothing can be described as "knowledge," concerning the effects of alcohol, which does not rest upon a large acquaintance with the results of somewhat recondite forms of chemical research, coupled with extensive clinical experience of the effects of alcohol in different healthy and diseased conditions, in different climates, in different doses, in varying degrees of dilution, in different combinations, and upon persons of different temperament. Knowledge of this kind would not admit of being condensed into an "arsenic, opium, and strychnia" formula; and any general condemnation or disapproval of the use of alcohol to which it might lead up would be expressed with qualifications of various kinds.

It must be admitted as beyond dispute that many generations of men, in many climates and countries, have derived pleasure from the consumption of alcohol, and that thousands of individuals have at least believed that, in addition to pleasure, they received some kind of help or sustentation from it in the performance of their daily tasks. The convictions hence arising are regarded as part of the accumulated experience of mankind, and they have been very little weakened by the knowledge, which has grown up alongside of them, that alcohol in excess is eminently prejudicial in a great variety of ways. Every sober consumer has regarded himself as affording a practical solution of the question how to obtain the benefits of alcohol while escaping from its injurious effects, and the guidance which the public now seek from the medical profession is mainly on the point of the possibility of use as distinguished from abuse. A denial of this possibility must rest upon much stronger grounds than mere denunciation; and, if it is to exert any influence upon human conduct, must be supported by a very large amount of evidence of a kind which can neither be contradicted nor explained away. It is useless to tell a man of business, who is abstemious throughout the day, and who enjoys a glass of Burgundy with his roast mutton, or a glass of port after his dinner, that he is consuming a functional poison, which acts first by stimulating and afterwards by depressing his nervous system. He will say that the stimulus is agreeable, that he is not conscious of the depression, and that his glass of wine adds to his sensations of comfort and of well-being. If he be told that it is comparable to arsenic, opium, or strychnia, he will be amused rather than convinced; and the character of his reply will be mainly governed by the degree in which he has cultivated politeness. It is at least premature in the present day to denounce the moderate use of alcohol by the community at large, and to rest the denunciation upon any basis of assured medical knowledge. The most promising sphere of activity, for any medical man who has convinced himself of the injurious effects of even moderate consumption, is to be found in his influence over individuals. If he will prescribe abstinence to patients in various conditions, carefully watching its effects upon the appetite, the secretions, the digestion, the sleep, and the immediate or deferred capacity for intellectual or physical exertion, he will acquire data of great present value in their applicability to the individuals concerned, and of great prospective value in their applicability to the race. It must be in this way, and not by platform denunciation, that the truth concerning the dietetic effects of alcohol will be made manifest, and will be rendered available for the general advantage of mankind.

